# Data Shepherding in Nanotechnology. The Exposure Field Campaign Template

**DOI:** 10.3390/nano11071818

**Published:** 2021-07-13

**Authors:** Irini Furxhi, Antti Joonas Koivisto, Finbarr Murphy, Sara Trabucco, Benedetta Del Secco, Athanasios Arvanitis

**Affiliations:** 1 Transgero Limited, Cullinagh, Newcastle West, V42V384 Limerick, Ireland; Finbarr.murphy@transgero.eu; 2 Department of Accounting and Finance, Kemmy Business School, University of Limerick, V94T9PX Limerick, Ireland; 3 Air Pollution Management, Willemoesgade 16, st tv, DK-2100 Copenhagen, Denmark; joonas.apm@gmail.com; 4 ARCHE Consulting, Liefkensstraat 35D, B-9032 Wondelgem, Belgium; 5 Institute for Atmospheric and Earth System Research (INAR), University of Helsinki, PL 64, FI-00014 Helsinki, Finland; 6 Institute of Atmospheric Sciences and Climate (CNR-ISAC) Via Gobetti 101, 40129 Bologna, Italy; s.trabucco@isac.cnr.it (S.T.); b.delsecco@isac.cnr.it (B.D.S.); 7 Environmental Informatics Research Group, Department of Mechanical Engineering, Aristotle University of Thessaloniki, 54124 Thessaloniki, Greece; tharvanitis@meng.auth.gr

**Keywords:** nanotechnology, exposure assessment, field campaigns, FAIR data, data management plan

## Abstract

In this paper, we demonstrate the realization process of a pragmatic approach on developing a template for capturing field monitoring data in nanomanufacturing processes. The template serves the fundamental principles which make data scientifically Findable, Accessible, Interoperable and Reusable (FAIR principles), as well as encouraging individuals to reuse it. In our case, the data shepherds’ (the guider of data) template creation workflow consists of the following steps: (1) Identify relevant stakeholders, (2) Distribute questionnaires to capture a general description of the data to be generated, (3) Understand the needs and requirements of each stakeholder, (4) Interactive simple communication with the stakeholders for variables/descriptors selection, and (5) Design of the template and annotation of descriptors. We provide an annotated template for capturing exposure field campaign monitoring data, and increase their interoperability, while comparing it with existing templates. This paper enables the data creators of exposure field campaign data to store data in a FAIR way and helps the scientific community, such as data shepherds, by avoiding extensive steps for template creation and by utilizing the pragmatic structure and/or the template proposed herein, in the case of a nanotechnology project (Anticipating Safety Issues at the Design of Nano Product Development, ASINA).

## 1. Introduction

The incorporation of nano-objects (NOs) (https://www.iso.org/obp/ui/#iso:std:iso:ts:80004:-1:ed-1:v1:en:term:2.5 (accessed on 5 June 2021)) to an increasing number of nanostructured materials (https://www.iso.org/obp/ui/#iso:std:iso:ts:80004:-4:ed-1:v1:en (accessed on 5 June 2021)) worldwide is driven by the benefits of novel applications [1,2]. The possible health effects derived from the exposure to these new NOs are still ambiguous [3]. Hazard identification and exposure assessments (occupational and environmental) are essential steps in a risk assessment and management framework. The highest potential risk for human exposure to NOs occurs in the workplace [4]. NOs are stored and handled in workplaces that span from research to production, usage and applications in different processes [5,6,7]. NOs are also unintentionally formed in various work places as Viitanen et al. [8] have shown with a literature review of 72 publications. As with all materials, validated control and monitoring of workplace exposure must be implemented and verified to protect the workforce [9]. A three-tiered approach has been described for occupational exposure assessment [9], which can be part of a risk mitigation strategy. Briefly, Tier I gathers information about process, materials and exposure to determine whether additional assessment is required. Tier II conducts a basic exposure or release assessment using a straightforward approach with easy-to-use portable equipment for release-related investigations and for real-time monitoring. Tier III obtains diverse information on airborne NOs to (a) determine whether exposure has the potential to occur, (b) quantify the level of exposure, and (c) determine the need for additional risk management steps.

Field Exposure Monitoring (FEM) campaigns produce a significant amount of data that are valuable for emissions, exposure and, subsequently, risk assessment. FEM data can also provide insights into the factors affecting process emissions, exposure levels and risks. These insights can be used to quantify the safe conditions of storage and use of NOs and identify efficient risk control techniques [10]. FEM data can be divided into three main categories related to process and material parameters, environmental conditions and worker behavior. Properly collected exposure data can be further refined with mechanistic models to estimate process emissions, emission control efficacies and dispersion of pollutants, along with other exposure determinants depending on the type of measurements. Quantifying emissions according to the process parametrization makes it possible to predict the process impact on work area concentration under different operational conditions. This allows conditions of use for any work scenario to be set by using a probabilistic exposure model [10]. This is currently the most promising approach for setting evidence-based conditions of use for processes and materials in the supply chain, which is one of the objectives in the update of REACH chemical legislation (DG ENV B2, DG GROW F1 “D. Evidence Base, Data collection and Better Regulation Instruments”) [11]. However, results from FEM studies are often not comparable to each other due to different methods and metrics [4], as a measurement standardization was recently developed for NO inhalation exposure assessment (CEN EN 17058:2018). The measurement metric (number, surface area, mass) by which the limit should be quantified is unclear specifically because exposure limits are not established for the majority of NOs. The fraction of background particles associated with the NO concentration is usually not well-known and it is ambiguous which parameters are best associated with biological responses [12]. Those uncertainties result in limited and heterogeneous formats of data reporting, which hinders the comparison, validation or integration of data [4,8,13]. High-quality data from exposure studies are needed to understand the exposure determinants, which are critical for occupational exposure model development [14]. While developing our template for the ASINA project, the GRACIOUS project (EU H2020, EC-GA No.760840) released templates for release, fate and exposure data collection [15]. Although sharing a number of concepts and determinants, some key differences will be noted in the discussion part.

Data are rarely leveraged beyond their original intended purpose, not only in academia, but in industry as well [16]. Fundamental enablers of digital transformation are technologies that deliver data that adhere to the FAIR principles [17]. FAIR data are considered key components of the new EU Industrial Strategy, Chemicals Strategy for Sustainability and Circular Economy Action Plan driven by the EU Green Deal approach [18], aiming to foster the Safe (and Sustainable) by Design approaches and allow for safe and sustainable innovation [18,19]. FAIR principles were coined in 2014 as a set of minimal guiding rules and practices for research data stewardship in the life sciences [20]. The European Union has released FAIR data management guidelines for Horizon 2020 projects [21] and any Horizon 2020 project that produces, assembles, or processes research data should provide the Data Management Plan (DMP) as an essential deliverable. As an overall practice, data management is connected with the entire lifecycle of data implementation, including the primary steps of data creation, capture, variations and final storage [22]. DMPs facilitate the above aspects, as they play a major role in data FAIRification. As part of making research data FAIR, a DMP should consist of information on:

- The handling of research data during and after the end of the project;
- What data will be collected, processed and/or generated;
- Which methodology and standards will be applied;
- Whether data will be shared/made open access;
- How data will be curated and preserved.

Previous articles have gone into great detail about FAIR′s four higher principles [17,20,23,24]. FAIR data principles have been developed to define good practices in data management. What constitutes “good” data management or stewardship is, however, still not clearly defined, and is generally left as a decision for the data creator and/or owner [23,25] and for a specific field of study. Recently, Papadiamantis et al. [26] have defined and proposed the scientific FAIR principles to complement the initial FAIR principles, and assist data creators on the steps needed to capture their data in a FAIR manner as they are produced.

In this paper, we describe the creation of a template for managing field monitoring data by following the execution of the proposed framework, describing the steps taken. We reveal the variables’ selection, which serves the FAIRification of exposure monitoring data and facilitates the evaluation of exposure determinants through modelling. Due to numerous combinations of processes and work environments, there are no individual measurement protocols applicable for all conditions. Thus, the focus is on the most important elements of field campaigns and minimum data requirements that data creators should report, which also enables data analysts to validate and reuse them. We show how the new role of data shepherd (see: [26] for shepherd’s definition) is involved across diverse fields with the goal to capture data in a reliable and comprehensive way. We describe the inner communication process between stakeholders, which allowed the generation of a co-created template, demonstrating the importance of involvement of all related partners, including data management. The main actors and their responsibilities related to data management are also defined. The template assists the integration of studies, the comparison of working environments and process-related descriptors. The template and guidance are beneficial for exposure assessors to produce and register relevant exposure data by following the FAIR and scientific FAIR principles. This helps researchers and the industrial community to capture relevant information for nanomanufacturing processes. The reporting guidance also helps the professionals involved in the data management process by showing a realistic pathway to template creation and data curation. The paper enriches the scientific community with valuable practical knowledge. The article is also beneficial for data shepherds; since this profession is still new, such detailed works help it to evolve in the right direction.

## 2. Materials and Methods

**Figure 1:**
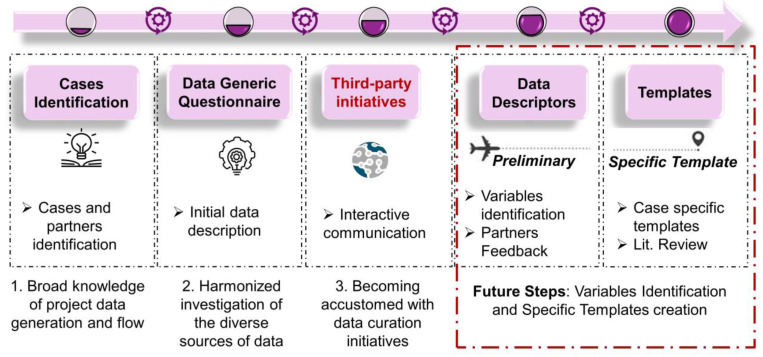
The roadmap towards the creation of case-specific data capturing templates for FAIRification purposes. In this article, we focus on the last two steps (red box) implemented for the field campaign exposure template. A detailed explanation of the previous steps can be found in the aforementioned article.

In the initial article published in the series of data shepherding in nanotechnology, Furxhi et al. [27] visualized the roadmap towards the creation of case-specific data capturing templates for FAIRification purposes, (see Figure 1) in the Horizon 2020 (H2020) ASINA project, with the guidance and support from the Transnational Access program of the H2020 NanoCommons project. The authors identified data cases, acquiring a broad knowledge of data generation and the flow within to meet project goals. Several data will become available from different partners (for data pillars in ASINA, see the aforementioned article).

In this paper, we demonstrate the progress from the proposed framework into the actual realization (template creation), focusing on FEM measurements, with the help and guidance of the data shepherd. Here, the objective of data collection is for logging conditions of use for occupational processes regarding synthesizing, handling or formulating nanostructured materials in the ASINA supply chain. The manufacturers handling and formulating these materials were identified and an initial questionnaire was distributed among the stakeholders. We show how we move from the questionnaires of initial data description circulated among the data creators, (see Section 3.1) to the usage of the preliminary template (see Section 3.2). We demonstrate the results of the inner communication process among partners regarding minimum data requirements and the selection of variables that led to a consensus within the project, and a template co-created with all stakeholders (see Section 3.3). In addition, we annotated the dataset to increase interoperability (see Section 3.4).

## 3. Results

### 3.1. Initial Data Description—Questionnaire

A questionnaire to provide a description of the data to be generated is circulated among data creators and analysts. The questionnaire aids the generation of preliminary templates by providing an initial representation of the data to be captured. The responses of data creators and analysts regarding FEM measurements are provided in Table 1. The questionnaire helps the data shepherd to structure and guide the communication between data creators and relevant involved stakeholders, to identify partners’ responsibilities, objectives and expected outcomes, and transfers an overview of needed resources.

The responses from data creators require information regarding the physicochemical characteristics of the NO and their amounts, pilot plants and room characteristics. The outcomes are derived from sampling stations using diverse instruments and monitors to obtain real-time particle number (and distribution), and sampled filters for offline analysis. Data analysts will employ probabilistic exposure models to measurement data for exposure determinants’ identification and quantification. Thus, in addition to experimental data, modelling data requirements are implied here but not yet explicitly mentioned. Whether and how measurements of concentration levels at relevant locations will discriminate between sources (background, process or other indoor sources) are not captured at this stage.

### 3.2. Preliminary Template from NIKC

NanoCommons’ suggestions for data capture include the CEINT NanoInformatics Knowledge Commons (NIKC) Excel spreadsheet [28]. The CEINT-NIKC template was modified (simplified and streamlined via the H2020 NanoCommons infrastructure project) for use by the H2020 project NanoFASE, to capture complex mesocosm experimental data. Spreadsheets are a common format used by most professionals of all subfields of science and, due to their simplicity and familiarity, are a reasonable choice for data entry format. For information related to the template, please refer to [27].

**Figure 2:**
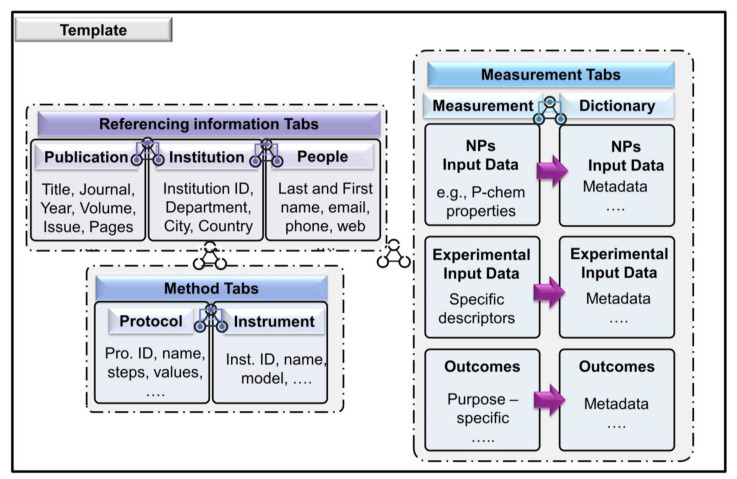
Dataset visualization. Tabs and variables included in each main tab.

In this article, we focus on the Method and Measurement Tabs (Figure 2). The other tabs of the worksheet (see Supplementary Materials, Template) contain referencing information related to data creators (People and Institution), or information related to any publication where data have been used (Publication). The Method Tabs contain Protocol and Instrument tabs that capture knowledge regarding the instrumentation and protocols related to sample preparation for offline characterization analysis when relevant. The Measurement tab is where information (measurement matrix) related to the process, environmental descriptors, work shift information, NO and matrix is captured. The main data recorded are location-based time series of airborne measurements performed during the manufacturing process. Finally, the Dictionary tab contains a detailed schematic metadata description and ontological annotation of the descriptors.

### 3.3. Literature, Inner-Communication and Descriptors Identification

**Figure 3:**
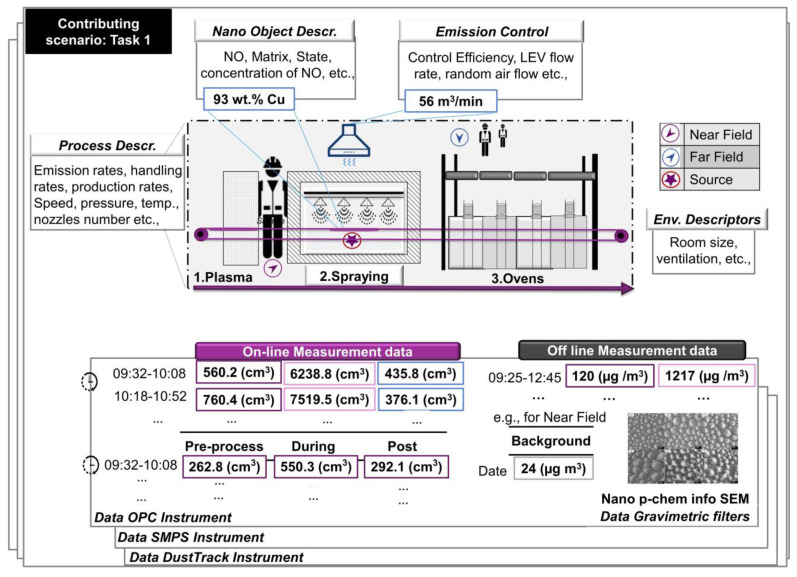
Visualization of exposure and emission data generation. The figure demonstrates a snapshot of data that are generated in one process, specifically one contributing scenario of an extensive exposure assessment.

Having at hand the data outline from the questionnaires and the preliminary NIKC template, a literature search and multiple experts’ feedback are essential in order to merge the two sources in revealing the present variables of importance while maintaining simplicity. This step is the most challenging part in the DMP and FAIRification processes, where the data shepherd and the stakeholders acquire spherical knowledge in the multidisciplinary fields of exposure assessment and modelling. The variables’ significance is defined based on stakeholder’s expertise and needs to reach project-specific goals. For example, particle size distribution can be applied to calculate the deposited dose during inhalation and to estimate particle deposition losses onto workplace surfaces and filter filtration efficiency. For predictive exposure assessment, particle emission rate from the chamber to the room is a requirement entailing information of air flow rates, besides the concentration measurements. Predictive exposure assessment requires knowledge of the exposure determinants, while source characterization requires measurements of concentration according to the process parametrization and analysis of mass flows [10]. Data availability and reliability following a tiered approach defines the precision of the modelled emission rates. Limited data quality results in precautionary emission rate estimates, higher exposure estimates and more stringent conditions of use. Data reporting requirements (Tier II/III) for data creators for each assessment [9] include information on emission sources, confounding factors, record of workplace activities (see Section 3.3.1 and Section 3.3.2), time series of workplace concentrations linked to the process, background concentrations and off-line analyses for chemical and/or morphological information (see Section 3.3.4) and information regarding instrumentation (see Section 3.3.5). To visualize the above information that the template captures, we compile the requirements for a demonstration scenario for spray coating machinery (Figure 3).

#### 3.3.1. Work Shift and Process-Related Descriptors

A comprehensive FEM requires the quantification of exposure along the process and work-related time intervals [3], which requires process-specific concentrations, e.g., [29,30]. In order to do this, work shift descriptors are captured to describe the contributing scenario in a task-specific manner. In cases where the production process is continuous, this information is less relevant (Table 2). Process definition is used to capture the general identification of a process using variable terminology in alignment with ECHA R.14 [31] requirements for exposure assessment, e.g., Life Cycle Stage (LCS), Sector of use (SU). ECHA descriptors are limited to main industrial process categories and may not always reflect the scenario for NO exposure scenarios. Therefore, we used ECHA descriptors selectively and propose, along with the workplace observations, a visualization (sketch, technical drawing, photo) as an essential aid in comprehensibly demonstrating the entire process and scenario to the data users (see Supplementary Materials, Measurement Tab).

Generic process descriptors such as automation, duration, emission rates (if available) and production rates are needed to estimate exposure times, emissions according to process parameters, production volumes (work intensity) and dispersion of the pollutants at the workstation (Table 3). ASINA specifically includes diverse processes (synthesis, spray coating, microemulsion, spray freeze drying, dip coating and padding, screen printing and cosmetic formulation) that have to be captured. Process parameters capture the variables that can affect the emission rate or dispersion of pollutants. Primary factors, such as process temperature, speed and pressure, are generic and should be included. The user can clarify the speed (roll speed, agitation speed of the emulsifying process) or temperature (fluctuation, or drying temperature during thermal curing) of relevance to be included. Secondary factors, which are process-specific, for example, in spray processes (Figure 3), information such as the number, arrangement and type of nozzles should be reported. Plasma treatment, on the other hand, is defined by the type and flow of treatment gas and the electrode type (ceramic or metallic). Those factors are inserted by the user according to the specific case. An example of data generated and capturing in this scenario are included in the Supplementary Materials.

#### 3.3.2. Emission Control and Environmental Descriptors

Emission control descriptors of the machinery are included in the template (Table 4). Local exhaust ventilation (LEV) efficiency plays an important role for concentration near the source when LEV is on. LEV can be designed so that it captures a fraction of emissions before entering the process room (e.g., fume hoods with 95% capturing efficiency). Measuring LEV concentration levels, extraction volume flow and filtration efficiency allow quantification of emissions to outdoor air or room air in cases of circulating LEV systems, where the air is returned to the room. The fraction that is escaped to the room can be specified from near field concentration and mixing of the concentration to the rest of the room by using balance principles.

Environmental descriptors are needed for the data analyst to estimate dilution and removal of pollutants from the process and other potential sources (Table 5). Separating process emissions from background emissions enables the calculation of process-specific exposure levels. Random air speed is a critical factor defining the mixing of emissions from near field to the rest of the room that can be measured by using, e.g., a hot wire anemometer. The random air velocity can be modified by process fans and temperature differences between process and room air and environmental conditions.

#### 3.3.3. Nanostructured Materials Descriptors

Data regarding the NOs used during manufacturing are required for exposure and hazard assessment. More importantly, this information enables data integration and traceability across the stages (NOs synthesis → incorporation to matrix → nanostructured materials) and is required for human and environmental hazard assessment [32]. Nanostructured materials descriptors (Table 6) are inspired by the particle emission library for articles and products containing NO [32], capturing information such as the category of the product in which the NO is incorporated, a description of the substrate, the NO used in the process (name, ID, core information), external and internal layer, the provider (partners within the project or external provider), and concentration in the matrix (e.g., mg of TiO_2_ per m^2^ of textiles). The form of NO in the matrix (embedded into the matrix, surface-bound, incorporated, impregnated) can be used to assess potential NO release in different life cycle stages.

#### 3.3.4. Time-Series Measurements

Measurements can be divided into on-line (Table 7) and off-line (Table 8) characterization. Concentration data are based on location, such as Breathing Zone (BZ), Near Field (NF), Far Field (FF), source, ventilation and process phase (post, during, pre). Those can be used to quantify the NO concentration where background (BG) concentrations from natural and incidental sources are subtracted [4]. Two common approaches to specify NO concentration levels are to 1) subtract pre- and post-process concentrations from process concentration levels or 2) measuring simultaneously at a co-location not influenced by the investigated process emissions and subtract the concentration from process concentration level [9]. Method 1 assumes that the BG concentration level is constant during the process and method 2 assumes that the co-location concentration represents the workstation location’s ventilation air concentration.

The units in the template defined depend on the detection technique. Common on-line particle sizing techniques are light scattering (e.g., optical particle sizer), particle mobility classification and single particle counting (e.g., scanning mobility particle sizer), particle aerodynamic classification and current induced by moving charge particle (e.g., electric low pressure impactor) [29]. It is important to specify the particle sizing technique because it affects the assumptions made during data analysis (e.g., unit conversions; see [29]. On-line personal exposure data are usually based on particles’ light scattering and diffusion charging because other portable on-line detection techniques are scarce [36]. From a modelling perspective, particle concentrations at different locations are used to calculate mass flows and further process specific particle emission rates in particle number, surface area, or mass. Furthermore, the concentrations can be exploited to characterize process-specific particle sources and estimate their influence [29].

Particle *size distribution* information is also added (Table 7), noting that, depending on size distribution and concentration, the deviation in the particle concentration number measured by the different instruments can be up to 50% [30]. For NO dust collection sampling, cassette wall losses should be reported; in conductive cassettes, the wall losses for respirable dust have been reported to be up to 56% as median wall deposits [37]. Size distribution reporting can be shortened with values of a fitted log-normal distribution that further simplifies the data reporting and applicability. Particle size information can be used for estimating particles’ deposition onto surfaces and to calculate the inhalation dose rates of deposited particles. The exposure levels and regional deposited doses during inhalation are used to estimate the inhalation exposure risk of process particle emissions [30].

On-line measurement techniques do not directly differentiate between agglomerates, aggregates and primary particles. Samples for offline analysis is performed to augment real time measurement (Table 8). Results from structural and chemical characterization (p-chem descriptors) of particles discriminate the sources to some degree [36]. Surface area analysis, e.g., by BET, can be used to help differentiate between agglomerates and aggregates [36].

#### 3.3.5. Instrumentation

Currently, there is no available instrument capable of meeting all the requirements of exposure characterization of airborne NO and there is insufficient comparability between measurement methods [38]. A suite of devices is typically used to conduct an exposure characterization [9]; thus, information regarding instruments is included. One tab is dedicated to instrumentation, covering the instrument name, the model, measured metric, detection limit, flow rate, etc., (see Table 9). The instrument is linked to the measurement tab for each row clarifying the source of the data. The data creator is responsible for correcting potential sampling line diffusion and dilution losses in sampling if not isoaxial and isokinetic sampling and reporting the correction methods.

Any protocols used during the campaigns are reported in the Protocols tab, i.e., sample preparation techniques of gravimetric assessment, or sample preparation for SEM/TEM analysis. In the case of FEM, any intercomparison results between instruments would be beneficial to be shown.

### 3.4. Annotation

Metadata are described as data about data and they are required in order for a dataset to be useful to others. Bringing nanodata together in a harmonized and interoperable fashion places demands on the description and curation of data [26]. There are ongoing efforts to encourage the reuse of data facilitated through, e.g., the NanoCommons project or the European Union Observatory for Nanomaterials (EUON) based on the requirements of the European Commission’s Open Data policy, which can succeed if complete (data can be found and understood without having to ask the data creators) high-quality data and metadata are available.

In accordance with the European Commission’s Open Data Policy and the FAIR data principles, the resultant template (metadata) is semantically annotated using established hierarchically structured domain specific dictionaries—ontologies. By offering a consistent terminology throughout a subject, ontologies are used to facilitate communication between people and organizations [39]. The most used nano-specific ontologies are the NanoParticle Ontology (NPO) and the eNanoMapper (eNM) ontology [26,40]. All descriptors in the measurement and instrument tabs are semantically annotated (see Supplementary Materials, tab Dictionary), to supplement the metadata. The descriptors can be annotated with several ontological terms, allowing the usage of numerous dictionaries to improve interoperability [40]. We combined and integrated different ontological IDs from diverse libraries when a single annotation from a source was missing.

The annotation increases interoperability and facilitates explicit understanding by providing a detailed metadata description to the dataset. In this way, the dataset becomes machine findable and interoperable with analogous datasets and supports indexing of data. The annotation requirements of the computational community are different from those of the regulatory community [26]. The current available data may be interoperable under a regulatory or scientific context but require processing to become computationally usable. Adding a comprehensive description is a key part that enables novel modelling and machine learning applications to be used and/or created by the nanosafety community.

## 4. Discussion

Clark et al. [41] noted that studies lack information on measurement strategy, and to date, establishing harmonized guidance is still pending. Harmonization is challenging because of the high diversity of processing techniques, materials and environmental conditions including emission and exposure controls. Various measurement strategies have been used within national projects, triggering interest in developing a harmonized approach to ensemble usability and comparability [8,9,36]. The template presented in this study works as a comprehensive worksheet, allowing users to import information regarding sampling locations and experimental details.

The template combines the information into tabs dedicated to measurements, instruments and protocols, is grounded on diverse sources [3,9,12,13,14,31,36] and offers an examination of both task-based emissions and airborne levels. Furthermore, the reliability of exposure models depends on the quality of user inputs. The templates facilitate data analysis and support the scientific community towards going FAIR.

Standardizing FEM data collection is, besides a scientific necessity, a business need, aiming at improving the use of data within insurers. By having more robust datasets for evaluating occupational exposure hazards, insurers are better positioned to provide guidance on how to control these hazards, thereby reducing adverse health effects, leading to fewer claims [42].

### 4.1. Responsible Persons and Data Use

Reliability, quality and applicability of data generation and capturing strongly depends on the communication among the data shepherd, the data creators and the data analysts [26]. Comprehensive exposure measurements are often challenging to reproduce because contextual information and metadata descriptions are missing. Communication and uncertainty analyses are often neglected as not as important as conducting laboratory experiments per se. The main responsibilities:

- For the data shepherd are facilitating the communication and understanding between the stakeholders of the process parameters, materials, working practices and the data to be captured.
- For the data creator are measurement of concentrations in relevant locations, evaluating the goodness of the concentration measurements, and reporting of the required metadata and underlying assumptions, such as identified sources and mixing of concentrations.
- For the data analysts are to link contextual information and concentration measurements, showing how the pre-process, process, and post-process levels are associated with the work activity to present assumptions in mass flow analysis, to evaluate links of regional concentrations and personal exposure and to report/justify uncertainty.

The metadata description and annotation part increase the interoperability and re-usability as per the FAIR principles, as they explain the context under which the data were captured [43]. At the same time, the inclusion of sufficient metadata allows for potential re-users to decide whether they can use the data under contexts different to those originally intended [26]. We combined and integrated different ontological IDs from diverse libraries (such as Exposure Ontology (https://bioportal.bioontology.org/ontologies/EXO (accessed on 5 June 2021)), eNanoMapper (https://bioportal.bioontology.org/ontologies/ENM (accessed on 5 June 2021)), Ontology of Consumer Health Vocabulary (https://bioportal.bioontology.org/ontologies/OCHV (accessed on 5 June 2021)), NanoParticle Ontology (https://bioportal.bioontology.org/ontologies/NPO (accessed on 5 June 2021)), PLOS Thesaurus (https://bioportal.bioontology.org/ontologies/PLOSTHES (accessed on 5 June 2021), etc.) when a single annotation from a source was missing [44]. A harmonized annotation including all descriptors is needed so that users can accurately identify and describe the processes causing emissions. Armiento et al. [39] demonstrated a two-step approach for ontologies extension using NanoParticle and eNanoMapper ontologies that lead to the addition of new concepts. The authors revealed that one issue that arises around ontologies is the degree of granularity usage during interpretation. A single schema focusing on reporting data elements is missing and it necessitates community consensus and the formulations of detailed recommendations on the metadata reporting requirements and well-defined words.

### 4.2. Comparison with GRACIOUS Data Collection Sheets

Recently, the GRACIOUS project (EU H2020, EC-GA No.760840) released templates for release, fate and exposure data collection [15]. Despite sharing a number of concepts and determinants, some key differences should be noted:

− We dedicate a tab for the user to insert protocol information used during the sampling such as for TEM or SEM offline analysis, or the sampling of gravimetric filters. An increased availability of validated protocols is necessary to advance both computational studies and material characterization through homogeneous data [45,46]. Sample preparation (such as duration of sample preparation, and solvents use) is an important step of any chemical analysis. The data creator is responsible to report relevant steps in the sample collection and analysis.
− Regarding the challenges of FAIRification, research institutions are often not sufficiently equipped with the specific knowhow and a number of researchers perceive these process requirements as additional paperwork that does not directly benefit their work [27]. Thus, the presented data collection sheet puts emphasis on the most essential elements to be captured, bypassing intense data reporting.
− The GRACIOUS template requires detailed p-chem characterization—for example, the moisture of NOs or aspect ratio. Moisture content alone cannot be used to predict environmental effects, i.e., the powder dustiness, because storing conditions also affect the dustiness [47]. NOs’ length quantification is currently not feasible at sufficient accuracy from workplace air samples; only indicative dimensions and concentrations can be provided [7,48]. Thus, we focus our p-chem characterization data on emission determination of offline analysis and not as an integral part of material characterization per se [4]. The difference refers to timing; the GRACIOUS template focuses on the p-chem of NOs before they are incorporated into the matrix, while we are interested in the characterization of released airborne NOs from offline analysis. Measurements and analyses not only need to be standardized, but also need to be made simpler, feasible and to some extent, cost efficient [12].
− A detail metadata description is provided to report the sampling strategy, measurement data and characterization part in an annotated manner to increase interoperability.
− Some variables not found in the GRACIOUS template include random air speed, local control efficiency and production rate (work intensity). Random air speed related to the process should be considered when estimating the dispersion of particles between NF and FF. LEV integrated in the process machine should be evaluated separately (e.g., when air is returning to room or exhausted outdoors, etc.). In addition, we invite and encourage data creators to add pictures and visualization files to help the next users, such as schemes of process and location of instrumentations. This helps data users to apply the data accordingly for their purpose.
− A FEM template should be flexible enough to include not prescribed factors that are process-specific, such as speed, temperature, pressure, the number of nozzles during a spray coating process or the ink viscosity in screen printing processes. This should allow the user to adjust the template in a process-specific manner.
− Concentration and emission data should be kept distinguishable. Emission depends on process parameters, while concentration depends both on emissions and environmental descriptors. We divide the process information-related data with the measurement requirements in clear and simple ways. We also limit the template to the FEM data without projecting assumptions to capture routes of exposure or potential of inhalation and dermal exposure. We provide a bottom-up designed template close to the raw data of exposure and emission assessments.
− NO fragments’ properties depend on the release process described in the release experiment. We do not capture release experimentations. Products have intentional use applications, and the emissions are based on those. For example, Ag-NPs applied to textiles for clothes and toys have leaching to washing liquid, sweat and saliva as relevant release pathways but textiles for outdoor use have weathering (UV, abrasion, leaching) as a relevant release pathway. Thus, a template dedicated to release emissions and characterization of released fragments is more suitable.
− GRACIOUS templates extend to regulatory requirements of chemical risk assessment. ECHA descriptors are designed mainly for traditional industry where the process categories (PROCS) can be difficult to associate with nanotechnology industry processes. Those features were not included in our template. The main ECHA descriptors are article category (AC), exposure scenarios (ESs), and PROCs. These are combined to contributing activities (CA) that are used to describe release from an AC in specific ESs by different PROCs. The CA, ES, and PROC descriptors with the quantitative NO release can be used for a “Lifecycle release and exposure” grouping strategy and parameterization of exposure and release models. AC defines where the substance has been processed (e.g., wood articles, plastic articles) and we maintained this information as the most relevant to the field campaign purpose. However, the categories are not well specified considering release. For example, ECHA AC1 is “Vehicles”, which is difficult to estimate where or for which vehicle the substance has been processed. Better sub-categories are needed which can be further grouped based on, e.g., matrix properties that behave in similar way under certain release circumstances (e.g., AC13 plastic articles: AC13.1 poly(methyl methacrylate), AC13.2 polypropylene, AC13.3 polyamide).

### 4.3. Comparison with the eNanoMapper and NECID Database

The eNanoMapper database provides a FAIR-aligned Nanosafety Data Interface (https://search.data.enanomapper.net/ (accessed on 5 June 2021)), with an aggregated search application to find data across diverse database instances [49]. The NanoReg2 database, included in eNanoMapper, is freely available and includes twelve datasets comprising of occupational exposure data derived from the Nano Exposure and Contextual Information Database (NECID) (https://perosh.eu/repository/necid-demo-version/ (accessed on 5 June 2021)) database. The data reporting format is not homogenized, and the structure reasoning is not reported. Data are missing and metadata description is not provided (see Supplementary Materials, Nanoreg2). For the moment, the exposure data are not yet in a reusable format; however, tremendous efforts have been put into making data re-usable. NECID focuses on describing operational conditions and working practices at a highly contextual information level. It covers an extensive number of parameters and descriptors that are specific to the workplace, worker, and measurements. NECID accepts raw measurement data with information about the instruments and sampling methods. In ASINA, we consider that it is the data analyst’s responsibility to analyze the exposure determinants together with the data creator and partners. This ensures better communication and increases the data’s reliability. This also significantly simplifies data collection requirements and ensures direct applicability of the data by users.

The NECID database is missing some of the most relevant contextual information considering worker exposure, such as material process/handling rate, production rates, emission control efficiency and random air flow at workstations. For example, one of the most relevant exposure determinants is the random air flow at the workstation diluting the concentrations after LEV [10]. For LEV, it is relevant to know if replacement air is extracted from the workstation or supplied elsewhere. This has a significant effect on the workstation concentration levels, regardless of the LEV capturing efficiency. Without such information, the results are not easy to be used to extrapolate other situations with sufficient precision. NECID rely more on qualitative exposure taxonomy than quantitative. Qualitative descriptors should be avoided in data collection because they are subjected to the interpretation of the data creator and analyst and the user, unless clearly defined and standardized. Categorization is useful when data are not available or the relevance considering the exposure or exposure extrapolation is not significant. However, in categorization, quantitative ranges should be provided to avoid misinterpretation of the categories.

Jeliazkova et al. [49] provide a detailed description of the eNanoMapper interface, the data included within and the path towards FAIRifying data (challenges and recommendations). The authors stress the lack of structured data available for re-use purposes and the need for user-friendly reporting formats, complete metadata description, ontology IDs and a harmonized terminology. The authors also recognize that available data are poor for computational purposes. It should be noted that exposure data in eNanoMapper represent the minority of safety data (40 out of 123.695 studies in total), showing that there is still a long road to make the safety aspect of occupational environment visible in the collective picture of nanosafety. The terminology, metadata description and ontology used in this template could accelerate the community’s pace on the FAIR path.

### 4.4. The Data Shepherd

From a data shepherd perspective, the most challenging part was to leap from the outlined information of the questionnaire to the comprehensive state of a filled-in ready-to-use data template. Data creators, for example, did not mention the importance of differentiating the location of measurements and the challenges in combining exposure and emission assessment. Gaining understanding in different but adjacent fields demands multiple collaborations from diverse partners for the shepherd [27]. Data shepherding as a novel concept and role in the field of nanotechnology is just forming [26]. Confusion may appear that the shepherd is a mediator for a partner to request more measurements from experimentalists, which is not the case. Communicating the needs and requirements of each stakeholder in a simple and understandable way is the core of the role and distances had to be kept from data management and data generation requirements. The data shepherd does not interfere with the experimental design plan but facilitates the information flow and ensures proper data and metadata capturing.

Burdened with the overview of all the project data, general comprehension of the relevant fields, identification of data creators, analysts, spreading the awareness of the value of FAIR data, ontology search, efficient data description communication management, actual template consensus realization and FAIRification of the data, the shepherd is a full-time dedicated role. Successful data shepherding of a multi-partner scientific research project requires distinct functioning and resources.

## 5. Conclusions

The data and metadata curation topic, including its central role to nanoinformatics, workflow, data completeness and quality, has been the focus of multiple collaborative efforts and publications in the nano-field. Data shepherds have been introduced as a new role in the process of data FAIRification. We revealed the role challenges such as the multiple inner communications with a variety of stakeholders and the requirement of data overview understanding across several disciplines. We conclude that confusion of the new role as someone who defines the data requirements is perceived. It is essential to stress that the data shepherd does not interfere with the experimental design plan, and instead, streamlines information flow and ensures appropriate data and metadata capturing. All parties work together towards minimum, essential data requirements.

The paper enriches the scientific paradigm with valuable practical knowledge. We demonstrated the execution of a proposed framework, describing the steps taken in the development of a template for managing field monitoring data. The template and guidance are beneficial primarily for data creators, for reporting findings in a FAIR way and for data managers and analysts, for comprehending the actual processes behind the data and reducing data uncertainty. The ad hoc collaboration between stakeholders and the shepherd allowed the generation of a consensus template. The role of the shepherd without the support of all parties cannot be realized and, in this paper, we steer the role to evolve in the right direction.

We stress the fact that field monitoring data have rarely been a subject for re-use. This template can be enriched with studies from the literature and present the knowledge that exists in a systematic way. Future data creators and shepherds can capture data in a FAIR way. The templates must be dynamic, subject to differentiation depending on the goals of a project. Furthermore, accommodating extensive metadata in the template realizes the opportunity for collected data to serve additional purposes. Multi-exploitation is a significant scope of managing data FAIRly. The road towards creating effective FAIR templates can be readily adopted in other fields dealing with potentially hazardous materials.

## Figures and Tables

**Table 1:** Form for collection of information on each dataset from the relevant partners.

Field Monitoring Campaigns for Exposure Assessment in Pilot Plants		
Element	Response—Data Creators	Response—Data Analysts
Data Identification		
Dataset description	Assessment of process emission rates and setting conditions of use.	Design field monitoring campaigns to quantify exposure determinants and workers exposure.
Source	Experimental measurement data.	Computational data
Partner’s activities and responsibilities		
Partner owner of the data; copyright holder	Each partner is responsible for the data provided to the data set.	Each partner is responsible for the data provided to the data set.
Partner in charge of data collection/analysis/storage/related WP (s)	Data collection: Data creators and Shepherd. Development of measurement matrix with stakeholders and reporting plan.	Data analysis: Data analyst, Data creators and Shepherd. Bridging information.
Expected input variables		
Description of the information required (WPs and/or Tasks) in order to move forward.	-NOs used in the processes and their use amounts per task/shift. -Pilot plants characteristics: dimensions, aspiration hood (position, size, flow rate).	Design of the measurement’s matrix with contextual information.
Expected outcomes		
Description of the specific endpoint measurement variables/outcomes.	-Particle concentration. -Particle size distribution. -Samples for off-line SEM particle characterization.	-Quantification of workstation concentrations, exposure levels, and other relevant exposure determinants.
Standards		
Detailed description of the methods/protocols	Set up of an experimental sampling station composed of: -SMPS to obtain the particle size distribution from 0.01 µm to 0.7 µm. -Optical Particle Counter to obtain the particle size distribution from 0.3 µm to 20 µm. -Monitors to obtain the LDSA (Partector-Naneos and MiniDisc-Testo) -Total filter samplers. Measurement standards (e.g., CEN EN 17058:2018; EN689)	-Analysis of mass flows, process emission rates and other relevant exposure determinants. -Validate mechanistic model against personal exposure. -Apply probabilistic model to evaluate exposure. -Set conditions of use.

**Table 2:** Work shift descriptors and process definition.

Category	Variables	Metrics	Metadata Description
**Work Shift Descriptors**	Task Identifier	Text	Short identifier, e.g., Task 1
	Use/Task name	Text	Task name, e.g., Powder pouring
	Description	Text	Task descriptions linked to process descriptors. If sampling is performed over multiple tasks each task should be described separately, e.g., Task 1.1 and 1.2
	Task duration	[min]	Task duration
	Time spent near process	[min]	Specify what is considered as near the source. For example, near the source is when distance is <2 m, for point emission sources and <10 m for diffusive sources (well mixed concentrations). The distances here are based on authors decisions.
**Process Definition**	Process Diagram	Picture (sketches, photos)	Process visualization including identified exposure determinants, such as source, process closures, LEVs and worker location.
	Life Cycle Stage (LCS)	List	LCS code according to ECHA use descriptor
	Sector of use (SU)	List	SU code according to ECHA use descriptor
	Process category (PROC)	List	PROC code according to ECHA use descriptor
	Description	Text	General description of the process, equipment, relevant process parameters, material chemical compositions and form (solid, liquid, gas). It is recommended to provide process machine manufacturer and model and material safety data sheets.

**Table 3:** Generic process descriptors captured in the template.

Category	Variables	Metric	Metadata Description
**Generic Process Descriptors**	Process parameters	Text	Factors that have an effect on the process emissions including materials. This is relevant for other users so that they can apply the process emissions reliably for their case. Data creators and partners are responsible for identifying relevant process parameters affecting the emission. Koivisto et al. [32] show some examples of relevant process parameters for specific processes and materials. Example: Temperature, speed, pressure. The user has the freedom to insert process parameters relevant to the case.
	Level of automation	List	If workers’ time use is not known, the level of process automation can be used to estimate the worker exposure time as: Fully—Workers are only involved in supervision and control walks. Worker exposure time < 30 min during an 8 h work shift. Highly—Very limited manual invention is required to run. Contact with the substance may be possible for a very limited duration. Worker exposure time between 30 and 90 min during an 8 h work shift. Semi—Manual intervention is repeatedly required, although large parts of the process are machinery assisted. Worker exposure time between 90 and 240 min during an 8 h work shift. Manual—This process requires an operator, who is conducting a task manually. Worker exposure time is over 240 min during an 8 h work shift. The time intervals here are based on authors’ selections.
	Process duration	[min]	Equal or smaller than task duration, depending on if the process is continuous or not. Process duration and rate should equal to the production amount during work shift.
	Material process /handling rate	[e.g., kg/min]	This describes the intensity of process during the task and should be expressed in units, such as: - Material transfer, handling or synthesis (e.g., kg/min; m^3^/min) - Surface treatment (e.g., m^2^/min) - Component production or processing (e.g., pieces/min, m^2^/min)
	Process emission rate/factor	[e.g., μg/min]	Process emission described in scalable quantities to calculate emission in different exposure scenarios. For example, emission factor can be expressed for surface treatment as mg/m^2^, material removal/additive processes as mg/kg, component production as mg/component, welding as mg/cm of welding, etc.
	Production Rates	[e.g., m^2^/min, pieces/min]	Similarly, as for emission rate, the production rate is described in scalable quantities.

**Table 4:** Emission control descriptors.

Category	Variables	Metric	Metadata Description
**Emission Control descriptors in process machine**	Emission control type	List	It is recommended to report the distance from the source and extraction funnel surface area for LEV and, for closures, the open surface area to evaluate LEV capturing efficiency (e.g., https://www.hse.gov.uk/lev/calculator.htm (accessed on 8 June 2021)). Emission control categorization helps to describe control techniques [33,34]: 1. Enclosure (automation): physical containment or enclosure of the source of emission; 2. LEV: exhaust ventilation systems located in close proximity of and directed at the source of emission; 3. Specialized ventilation systems: mechanical ventilation systems specifically designed for displacement of air contaminants in small, designated areas or systems intended to supply fresh air to workers; 4. Suppression techniques: techniques where an additive is added to an activity or process in an attempt to suppress emissions; 5. Segregation sources: isolation or segregation of sources from the work environment without containment of the emission source itself; 6. Worker separation: a personal enclosure within a work environment. It is recommended to provide the emission control’s manufacturer, model and efficiency. Note that emission control efficacies should be reported as well and in worker separation, the time that the worker spends in a separated area and close to the process.
	Local Control Efficiency	[%]	Efficiency is 100% for perfect local control capturing all emissions and 0% when it acts as a ventilation exhaust near the source.
	LEV flow rate	[m^3^/min]	LEV air extraction and exhaust locations should be specified (e.g., mechanically assisted LEV where replacement air is partially from outdoors or LEV circulating the room air through a filter).

**Table 5:** Environmental descriptors.

Category	Variables	Metric	Metadata Description
**Environmental descriptors**	Room size	[m^3^]	An approximate of the room volume where air exchange is considered to follow the room ventilation rate.
	Room ventilation	[m^3^/min or 1/h]	Ventilation by LEV should be treated separately from the general ventilation.
	Other possible emissions sources	Text	Emissions from other processes in the same room as the sampler can increase the concentration level. Incoming ventilation air may also transport pollutants depending on the ventilation design and location of the facility. All active processes during the task that could impact measurements should be mentioned.
	Workstation closure	Text	The workstation closure where the worker performs the task is used to estimate how much air mixing between near and far field is limited. This is relevant mainly for point sources. Workstation closure is expressed as a side of a cube/rectangle, where sides may be closed in combination of back, one side, both sides and top.
	Random Air flow at workstation	[m/min]	Random air speed gives information about pollution dispersion near the source [35]. Near is defined as the distance within the worker breathing zone and the source. Typically, this varies from 30 cm to 1 m.

**Table 6:** Nanostructured material descriptors.

Category	Variables	Metric	Metadata Description
**Nanostructured material Descriptors**	Article Category (AC) and sub-category	List	AC code according to ECHA use descriptors
	Substrate (Matrix type)	Text	Description of the matrix (Polyester fibers, Polymethylmethacrylate, clay, epoxy, fil, liquid) and chemical composition
	NO name and core	Text	Name, ID code (i.e., JRCxxx) CAS and or ES number and chemical composition, i.e., TiO2 pigment (93% rutile), (Tioxid TR81; CAS-Nr. 13463-67-7)
	External Layer	Text	NO name in surface layer 1 (more external layer)
	Inner Layer	Text	NO name in surface layer 2 (inner layer)
	NO Provider/Supplier	Text	NO manufacturer provider (partner related to the project ASINA) or supplier
	Concentration	[wt.%]	NO concentration in material mass unit, e.g., wt.%, µg/g, etc. Usually, this information is provided by a different partner, but the final concentration of NOs in a product is of paramount importance and should be captured when known.
	Form of NO in matrix	Text	State of NO (s) in the matrix/media: embedded into, surface bound, incorporated, impregnated, in liquid, dispersion, powder, solid, paste

**Table 7:** On-line Measurement’s descriptors.

Category	Variables	Metrics	Metadata Description
**On-line Measurements based on Location and Phase**	Collection Interval	hh:mm-hh:mm	Start–End Time of the measurement
	Instrument		Instrument name. Detailed information regarding instruments is inserted in a different tab (Instrument Tab)
	Ventilation	[m^3^/min or 1/h]	Incoming/outgoing ventilation air
	Source	Metric: P Numb (cm^−3^), Lung Deposited (µm^2^ cm^−3^), Size Distribution (dN/d(D_p_)), Mass (µg m^−3^) AND Variations: Average, Geometric mean (GM), Geometric standard deviation (GSD), Arithmetic mean (AM), Standard deviation (SD)	Very close to the source or at the source (e.g., tailpipe, inside the process closure or inside fume chamber). Report average and standard deviation
	NF		NF—Close to the source where pollution is mixed to the rest of the room. At working station, NF should cover the worker’s breathing zone and source, and provide the distance from the source.
	FF		FF—Typically 5 to 10 m from the NF measured in the same room (same room where the source is).
	BZ		Breathing zone measured within 30 cm from the worker’s nose and mouth (EN 689)
	BG		Other spaces where ventilation replacement air flows are potentially carrying pollutants to the process room. Background can be estimated from pre- and post-process measurements or by measuring background concentration from FF that is not affected by the source emissions.
	Pre-process		Pre- and post-process concentrations are one way to estimate the concentrations from the process. This shows how much the concentration is increased by the process, when assuming background concentration as constant (as average of pre- and post-concentrations). Specified for NF, FF, or source depending on the assessment objective (e.g., worker or co-worker exposure to process particles or emissions from the process, respectively); (2) Specified for any instrument. Local background particle exposure: local work area eight-hour time-weighted average particle number or mass concentration that excludes any contribution of particles from the nanotechnology process. This value is specific to each work environment. This value should be determined following repeated measurement of the particle number and mass concentration when the nanotechnology process is not in operation. The results of such measurement should be converted to an 8-hour time-weighted average value and the median of all values used as the basis for the recommended local particle reference value.
	Post-Process concentration		
	Process concentration		Concentration during the process.
**Log-normal size distribution Descr.**	Instrument		
	Location and Phase		NF, FF, BG, etc., Post-/During/Pre-process
	X	[N (cm^3^)]	P number concentration (>10 nm)
	GMD	[nm]	geometric mean diameter, AND information such as: particle diameter corresponding to 50% of the cumulative distribution
	GSD	[-]	geometric standard deviation, AND information such as: particle diameter corresponding to 84% of the cumulative

**Table 8:** Offline measurements descriptors.

Category	Variables	Metrics	Metadata Description
**Offline Measurements and Analysis**	Collection Interval	hh:mm-hh:mm	
	Instrument	Text	Instrument name. Detailed information regarding instruments is inserted in a different tab (Instrument Tab)
	Sample collection/description	Text	A description of the sample collection, e.g., gravimetric, surface dust sample
	Ventilation	[m^3^/min or 1/h]	
	Source	Metric: P Numb (cm^−3^), Lung Deposited (µm^2^ cm^−3^), Size Distri (dN/d(dp)), Mass (µg m^−3^) AND Variations: AVEG, SD, GM, AM, GSD,	Same as Table 7
	NF		
	FF		
	BZ		
	BG		
**Chemical Information**	Instrument	Text	I.e., ICP-MS, EDX
	E.g., Elemental composition (wt.%), Impurities, Crystallinity phase		Elemental composition, i.e., Major: Ti, O Minor: Si, Al, Zr, P, and organic coating
			Impurities: Any unwanted organic, inorganic, and residual solvents in drug substances and final products.
			The detection and quantification of the amount of amorphous material within a highly crystalline substance.
**Structural** **Information**	Instrument	Text	I.e., SEM, TEM, BET
	E.g., Pristine size (nm), Shape, Specific surface area (m^2^/g), Bulk density (g/cm^3^), Zeta potential (mV), Dissolution rate (mass/time unit), Solubility(g/L)		Specific surface area total surface area of a material per unit of mass, or solid or bulk volume
			Bulk density
			Zeta: The measurement of the overall charge a particle acquires in a specific medium
			Dissolution rate actual release rate of the compound at the given particle size, etc.
			Solubility: capacity of a solute to dissolve in a pure solvent

**Table 9:** Instrument descriptors.

Category	Variables	Metadata Description
**Instrument**	Instrument Name	e.g., FESEX, EDX, ICP-MS, OPC, SMPS, DustTrack, INSPEC, etc.,
	Model	e.g., Sigma, DISCMini, Partector, DusttRACK, Bravo, Pump
	Measured Metric (Outputs)	e.g., Mobility/aerodynamic/optical particle Number concentration (1/cm^3^), Lung Deposited Surface Area (µm^2^/cm^3^), Particle Size Distribution (dN/d(dp)), Particle Mass concentration (µg/m^3^)
	Detection Technique	e.g., light scattering, particle mobility classification and single particle counting, particle aerodynamic classification and current induced by moving charge particle
	Size Range	e.g., 30 nm–~700 nm, 250 nm–20 μm, 11.1 nm–1082 nm, >30 µg
	Detection Limits	Lower and upper detection limit
	Total Flow (L/min)	Instrument sampling flow?
	Manufacturer Information	e.g., Agilent technology, Santa Clara, USA. TSI, Shoreview, USA, Grimm Aerodol Technik, Airnring, Germany.

## Data Availability

The raw data files are being uploaded into the NanoCommons Knowledge Base (https://ssl.biomax.de/nanocommons/cgi/login_bioxm_portal.cgi (accessed on 5 June 2021)).

## Supplementary Materials

The following are available online at https://www.mdpi.com/article/10.3390/nano11071818/s1, S1: Template, S2: Nanoreg2 data.
